# Development and evaluation of serological screening based on one dried plasma spot for HIV, syphilis, and HCV

**DOI:** 10.1186/s12985-023-02225-6

**Published:** 2023-12-11

**Authors:** Jie-qiong Ma, Ya-nan Ren, Shi-yuan Wen, Ao-bo Dong, Wen-ge Xing, Yan Jiang

**Affiliations:** 1 College of Basic Medical Sciences, Shanxi Medical University, Taiyuan, China; 2 National HIV/HCV Reference Laboratory, National Center for AIDS/STD Control and Prevention, China CDC, Beijing, China; 3 Fangshan Center for Disease Control and Prevention, Fangshan, Beijing, China; 4 Third Hospital of Baotou City, Baotou, 014040 China

**Keywords:** Dried plasma spots, Human immunodeficiency virus, Hepatitis C virus, *Treponema pallidum*, ELISA

## Abstract

**Background:**

In the effort to prevent and control HIV/AIDS, China has established a national sentinel surveillance system. However, some sentinel sites face limitations in environmental resources and accessibility, prompting the exploration of alternative sample strategies. Dried plasma spots (DPS) samples are viewed as promising alternatives to traditional plasma samples due to their advantages, including sample stability, easy storage, and convenient transport. This study aims to develop a method for screening HIV, *Treponema pallidum* (TP), and Hepatitis C Virus (HCV) using DPS samples and assess their performance.

**Methods:**

Based on existing commercial assay kits, a detection method was established through the optimization of experimental parameters, including the amount of plasma on filter paper, the volume of elution solution applied to dried plasma spots, the size of dried plasma spots, elution solution volume, elution solution components, elution temperature, and elution time. A series of laboratory evaluation panels were constructed for laboratory assessments, including the laboratory basic panel, laboratory interference panel, and laboratory precision panel. Additionally, clinical samples were used for evaluation.

**Results:**

Optimal conditions for DPS sample extraction were: plasma volume, 100 µL; DPS size, whole spot; eluent volume, 500 µL; eluent, PBS with 1‰ Tween20; elution time, 2 h; elution temperature, room temperature. A total of 619 paired plasma/DPS samples were tested by both methods. The DPS-based ELISA method exhibited 100% sensitivity/specificity for HIV, 98.6%/100% for TP, and 99.6%/100% for HCV. Kappa values between the plasma samples and DPS samples were 100% for HIV, 99% for TP, and 100% for HCV. The DPS-based ELISA method failed to detect 1 HCV mono-infected sample and TP in 1 HIV/HCV/TP co-infected sample. For the HIV/HCV/TP co-infected sample, the S/CO in the plasma sample was 2.143 and in the DPS sample was 0.5. For HCV, the S/CO (sample OD/cut-off) was 3.049 in the plasma sample and 0.878 in the DPS sample.

**Conclusions:**

A single DPS, following one-time standardized processing, can be used to detect HIV, HCV, and TP. Researching and establishing laboratory testing methods better suited for China's sentinel surveillance have significant practical applications in improving HIV testing in resource-constrained environments.

## Introduction

The World Health Organization (WHO) recently reported that the total number of people living with Human immunodeficiency virus (HIV) has reached around 36.9 million, and 110 million persons are hepatitis C virus (HCV)-antibody positive. Furthermore, worldwide there are about 6 million new cases of syphilis per year [1, 2]. However, HIV, syphilis, and HCV are underdiagnosed, and most individuals are unaware they are infected. Moreover, public health diagnosis and prevention systems for HIV, syphilis, and HCV is inadequate as they are not provided on an adequate scale and services do not reach everyone in need, especially in low- and middle-income countries (LMICs), resource-limited settings, or difficult-to-access populations [2, 3]. To this end, the WHO has proposed many strategies and methods to increase testing, including innovative detection methods such as the use of dried blood spot (DBS) samples or dried plasma spot (DPS) samples for testing [4, 5].

DPS testing using filter paper as a vehicle for the storage and transportation of blood specimens does not require cold temperature storage as is needed for traditional blood samples [6, 7]. Furthermore, DPS samples packaged with a desiccant can usually be transported as a non-hazardous material via postal services, and samples can be sent to a laboratory that is in an urban area, far from a resource-limited setting [8]. Overall, DPS sampling is considered to be a convenient, feasible, and cost-effective alternative to using traditional blood samples [9, 10].

DPS are used for many biochemical assays, such as testing for specific enzymes, determination of metabolites, therapeutic drug monitoring including the quantification of mycophenolic acid (MPA), daptomycin, and phenyl glucuronide, and the diagnosis of infectious diseases such as HIV, hepatitis B virus (HBV), HCV, and tuberculosis [6, 11–14]. In the diagnosis of tuberculosis, the content of IP-10 and IFN-γ in dried plasma spots is tested, while in studies related to the detection of diseases such as HIV and HCV using dried plasma spots, the general practice is to test for relevant antigens, antibodies, or viral loads in the dried plasma spots [9, 12, 15, 16]. Differences in the detection factors may lead to variations in the sensitivity of infectious disease testing [6, 11–14]. DPS are used to test for HIV serological markers such as HIV-1 p24 antigen, for HBV serological marker including HBs Ag, HBe Ag, anti-HBc Ag, anti-HBs Ag, and anti-HBe Ag, and for the quantification of HIV-1 RNA, HBV DNA, and HCV RNA, and studies have shown that results obtained with DPS are concordant to those using plasma specimens [9, 15, 16]. However, few studies have examined the use of DPSs for simultaneously testing for anti-HIV, anti-TP, and anti-HCV antibodies.

The purpose of this study was to optimize the elution conditions of DPS, and develop and evaluate a DPS-based enzyme-linked immunosorbent assay (ELISA) using commercially available kits to detect anti-HIV, anti-*Treponema pallidum* (TP), and anti-HCV antibodies simultaneously using one specimen. Comparison analysis between DPS and plasma samples was done to evaluate the performance of the DPS-based ELISA method to simultaneously screen for HIV, TP, and HCV.

## Material and methods

### Sample collection

Plasma samples were obtained from the sample depository of the National Center for AIDS/STD Control and Prevention, Chinese Centers for Disease Control and prevention (CDC). The samples were used to construct a laboratory basic panel, a laboratory analytical specificity panel, a laboratory linear dilution panel, and a laboratory precision panel.

A total of 619 paired plasma/DPS samples were used for comparison of the DPS-ELISA method and traditional plasma sample testing. Of these, 150 paired plasma/DPS samples were collected from drug users between June 2018 and July 2018 at the Dehong Autonomous Prefecture in Yunnan Province, China. The rest were obtained from the National Center for AIDS/STD Control and Prevention depository.

## Sample collection and preparation

Blood samples were collected by venipuncture, and then were centrifuged to obtain plasma. Plasma was stored at − 70 °C until use. Based on the optimized parameters developed in this study, DPS samples were obtained by spotting a given volume of plasma onto Whatman 903 filter paper, and then drying at room temperature (RT) for at least 4 h before processing. The DPS samples were stored at − 20 °C until use. For testing, the DPS samples were punched or cut from the filter paper, and then immersed in a given volume of elution buffer and incubated for a specific period of time at a specific temperature (as determined in this research). The solution after elution was used for the assay.

## Detection of anti-HIV, anti-TP, and anti-HCV antibodies in DPS and plasma samples

Plasma samples were tested for anti-HIV, anti-TP, and anti-HCV antibodies using commercially available ELISA diagnostic kits according to the manufacturers’ instructions. They were Diagnostic Kit for Antibody to Human Immunodeficiency Virus (ELISA), Diagnostic Kit for Antibody to Treponema Pallidum (ELISA) and Diagnostic Kit for Antibody to Hepatitis C Virus(EILSA), respectively. Results were confirmed using a detection kit for antibody to HIV (1 + 2) (RIBA), the syphilis toluidine red untreated serum test, and a detection kit for antibody to HCV (RIBA). RIBA means that Recombinant Immunoblot Assay, which is similar to Western Blot (WB). The DPS eluates were tested with the same kits used to test the plasma. All these kits were purchased from WANTAI BioPharm, China.

## Optimization and establishment of the DPS-based ELISA assay

To identify the optimal elution conditions for the DPS samples, the DPS samples were eluted in using different experimental parameters. These parameters were: (a) volume of plasma used for the DPS sample (100 µL or 65 µL); (b) different combinations of the size of the DPS sample and the volume of eluent (DPS size; whole spot, 1/2 DPS, 1/4 DPS, 1 disc (6 mm), 2 discs (6 mm): eluent volume; 1 mL, 500 µL, 300 µL, 200 µL); (c) elution buffer (PBS with 1‰ Tween20, PBS with 1‰ TritonX-100, PBS with 1‰ Tween20, PBS with 1‰ TritonX-100); (d) different combinations of elution time and elution temperature (2, 4, and 6 h, and 4°C, RT, and 37°C). The selection of all the parameters mentioned above is based on a combination of literature review, preliminary experiments, and constraints such as the capacity of the filter paper.

## Laboratory evaluation of the DPS-based ELISA method

### Laboratory linear dilution panel

Ten HIV-positive plasma samples and 10 HIV-negative plasma samples were selected and mixed, respectively. Serial dilutions of the mixed positive plasma samples were diluted with mixed negative plasma samples (2^0^ to − 2^30^). Prepare 30 15-mL-centrifuge tube, number them 2^1^–2^30^ and add 5 mL mixed negative plasma into each tube. After that add 5 mL mixed positive plasma into 2^1^ tube and mixed thoroughly, then pipet 5 mL mixture from 2^1^ tube into 2^2^ tube; and repeat this process in order and get the serial dilutions samples. Matched DPS samples were also prepared with the same procedure. Plasma samples and matched DPS samples were tested in duplicate to determine the linear range of the DPS-based ELISA method, and optimize the DPS-based ELISA method for HIV. In order to analyze the effect of DPS sample volume of the HIV test result, 3 other serial dilutions were made as described above based on the linear range obtained for the DPS-based ELISA method.

The same procedures were performed for the analysis of TP and HCV.

## Laboratory basic panel

Fifteen HIV-positive samples, 15 TP-positive samples, 15 HCV-positive samples, and 45 samples there were negative for all 3 agents were selected and prepared as DPS samples to construct a laboratory basic panel to assess the sensitivity and specificity of the DPS-based ELISA method to screen for HIV, TP and HCV. The laboratory basic panel was constructed using the established DPS-based ELISA method.

## Laboratory analytical specificity panel

In order to evaluate the capability of analytical specificity of the DPS-ELISA method, 3 HIV/HCV co-infected samples, 3 HIV/TP co-infected samples, 3 HCV/TP co-infected samples, 3 HIV/HCV/TP co-infected samples, 3 HBV-positive samples, 3 HBV/HIV co-infected samples, 3 HBV/HCV co-infected samples, and 3 HBV/TP co-infected samples were prepared as DPS samples to construct the laboratory analytical specificity panel. The laboratory analytical specificity panel was constructed using the established DPS-based ELISA method.

## Laboratory precision panel

Based on the linear range of the DPS-based ELISA method to screen for HIV, TP, and HCV, 3 antibody levels (high, medium, and low) in the DPS samples were used to construct the laboratory precision panel. The laboratory precision panel was constructed using the established DPS-based ELISA method, 3 tests were run per day for 5 days. The precision was analyzed by calculating the inter-day, intra-assay, and inter-spot coefficients of variation (CV).

## Comparison between DPS samples and plasma specimens

A total of 619 plasma and DPS matched samples were obtained, and tested by the 2 methods. Plasma sample results were used as the reference standard. Results of the 2 methods were compared to determine the feasibility of the DPS-based ELISA method in for screening for HIV, TP, and HCV.

## Ethical considerations

The study was approved by the Ethics Review Committees of the National Center for AIDS/STD Control and Prevention, China CDC, and was performed in accordance with all relevant guidelines. All samples were delinked from identifying patient information before testing.

## Results

### Optimization and establishment of the DPS-based ELISA method

#### Volume of plasma in DPS samples

**Fig. 1:**
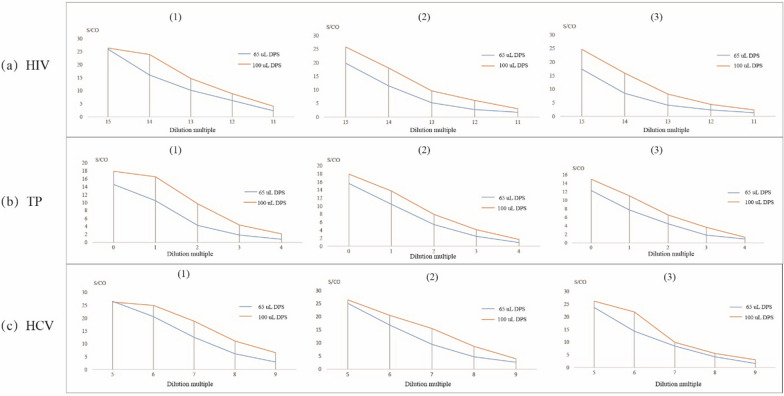
Comparison of ELISA tests for detection of anti-HIV/anti-TP/anti-HCV in DPS samples with different volume of plasma. Note: The figures in brackets represent different serial dilution

The effect of different volumes of plasma used for the DPS samples for anti-HIV, anti-TP, and anti-HCV testing is shown in Fig. 1. In all serial dilution experiments of all 3 agents, the test result curves (S/CO) using 100 µL of plasma were higher than that when 65 µL of plasma was used.

#### Size of DPS sample and volume of eluent

**Fig. 2:**
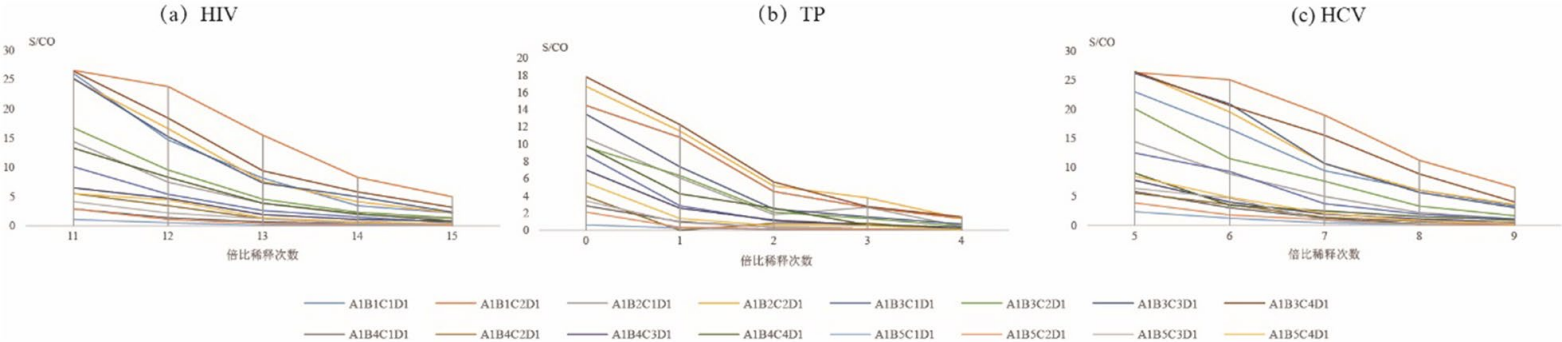
Comparison of ELISA tests for detection of anti- HIV/anti-TP/anti-HCV under different combinations based on size of DPS and volume of eluent Notes: **A** indicates that DPS sample contained 100 µL plasma; **B** represents the size of DPS sample [whole spot, 1/2 DPS, 1/4 DPS, 2 discs and 1 discs (6 mm)]; **C** show that the volume of eluent [1 mL, 500 µL, 300 µL,200 µL]; **D** means the amount of sample according to test kits

As shown in Fig. 2, for HIV and HCV the detection results (S/CO) using the combination of A1B1C2D1 were greater than those using other combinations of sample size and eluent volume (Fig. 2a, c). However, for TP, the detection results using the combination of A1B1C2D1 was lower than when the A1B3C4D1 combination was used (Fig. 2b).

#### Elution buffer

**Fig. 3:**
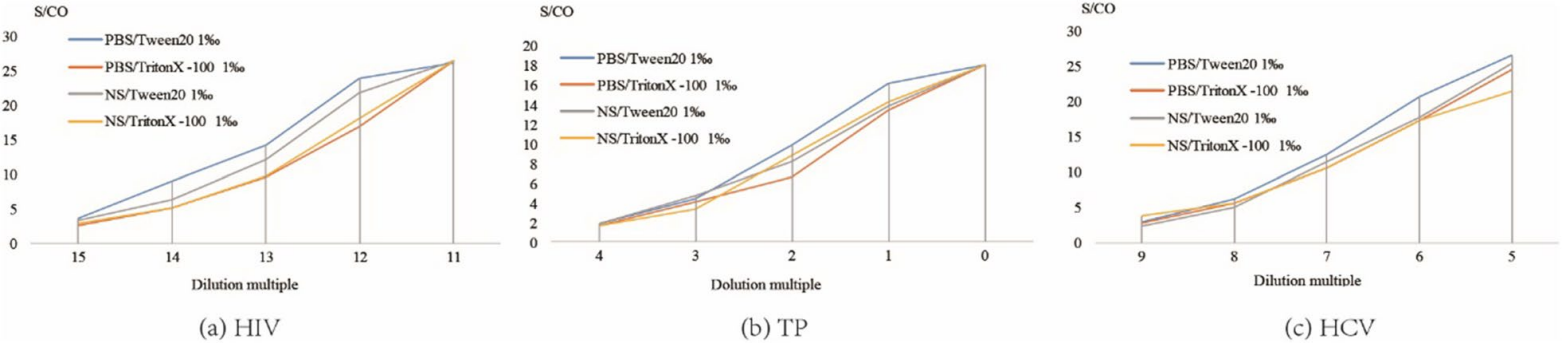
Different elution buffer effects on the DPS-based ELISA method for detection of anti-HIV, anti-TP and anti-HCV

As shown in Fig. 3, the detection results for all 3 agents were the highest when PBS with 1‰ Tween20 buffer was used.

#### Elution time and elution temperature

**Fig. 4:**
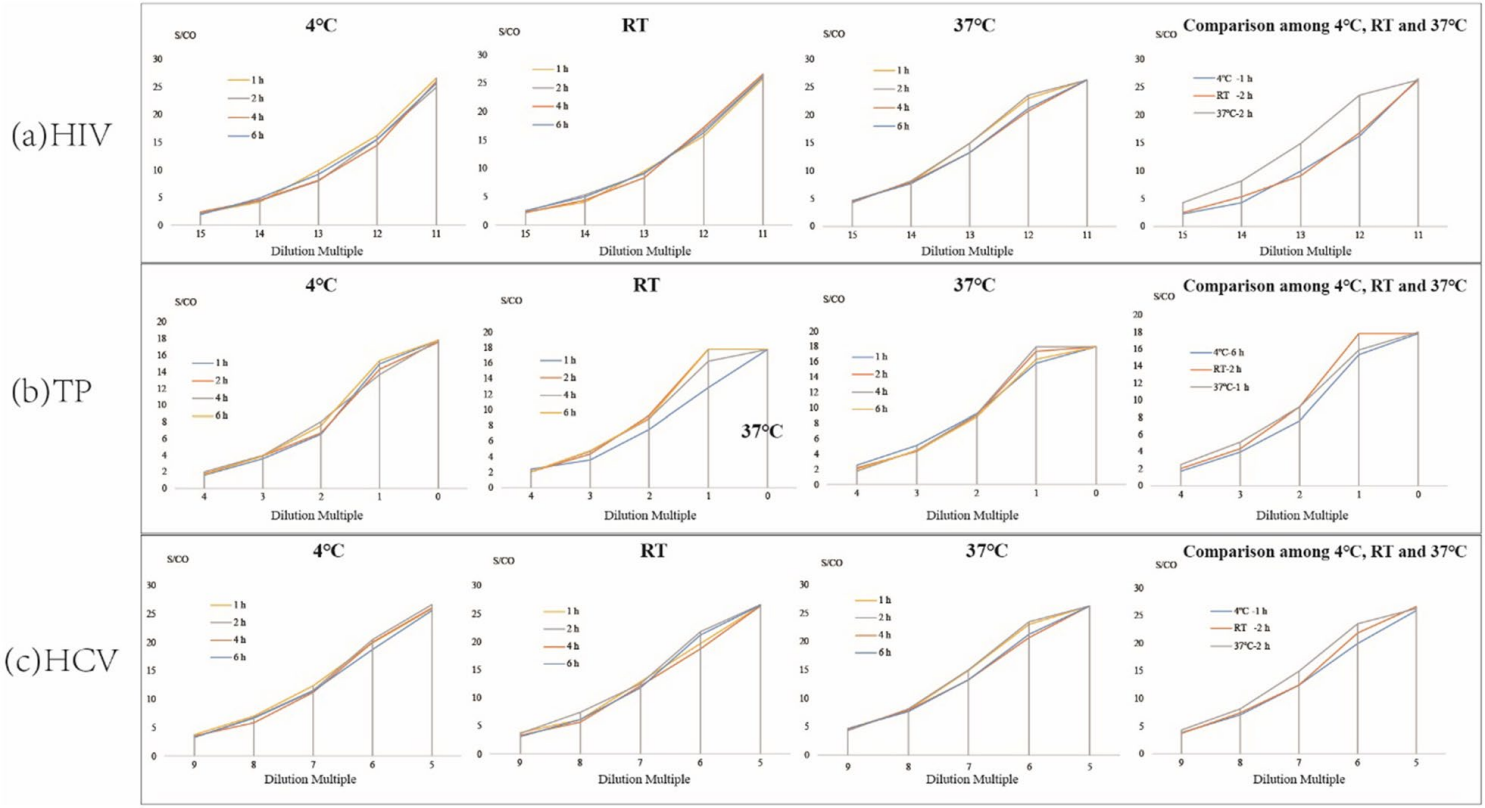
The variation of test results of anti-HIV, anti-TP and anti-HCV at different elution temperature with the elution time

Results of the effect of different combinations of elution time and elution temperature on the performance of the DPS-based ELIA method are shown in Fig. 4. For HIV, TP, and HCV the best elution time was 2 h at RT, or 1 h at 37°C. At 4°C, the best elution times were 1, 6, and 2 h for HIV, TP and HCV, respectively. Further analysis was then performed to determine the optimized combination for the detection of all 3 agents (Fig. 4a, b, c). The results indicated that elution for 2 h at RT provided optimal detection of all 3 agents.

#### Optimal conditions

*No statistically significant differences were observed*. Based on the aforementioned experimental results, the optimal conditions for the DPS-based ELISA assay to detect all 3 agents are**:** plasma volume of was 100 µL, whole spot DPS size, eluent volume of the elute was 500 µL, elute of PBS with 1‰ Tween20 buffer, elution time of 2 h, and elution temperature of RT.

#### Evaluation of the DPS-based ELISA method to screen for HIV, TP and HCV

In order to evaluate the linear range, sensitivity, specificity, precision, and anti-jamming capacity of the DPS-based ELISA method to screen for anti-HIV, anti-TP, and anti-HCV antibodies simultaneously, laboratory panels were constructed, and the DPS-based ELISA method was tested using these panels.

#### Linear range

**Fig. 5:**
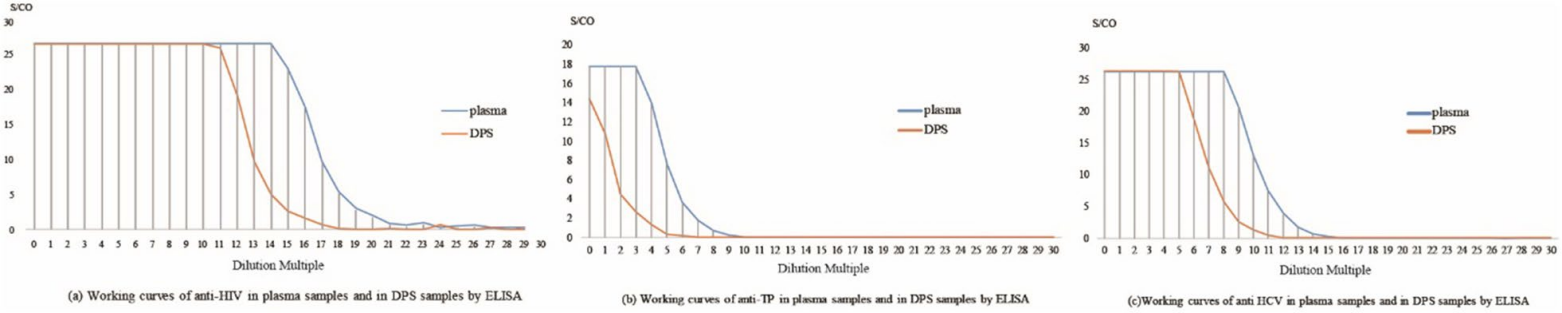
Working curves of anti-HIV, anti-TP and anti-HCV in plasma samples and in DPS samples by ELISA. Note: **a** has been published in Chinese Journal of AIDS & STD and used as a reference in this study

The linearity of the DPS-based ELISA method for anti-HIV, anti-TP, and anti-HCV is shown in Fig. 5. The linear range of determination for anti-HIV, anti-TP, and anti-HCV was 2^14^ to 2^19^ (R^2^ = 0.98), 2^3^ to 2^7^ (R^2^ = 0.97), and 2^8^ to 2^13^ (R^2^ = 0.98), respectively. As shown in Fig. 1, for the detection of anti-HIV, the DPS-based ELISA method was linear in the dilution range of 2^11^ to 2^15^, with a correlation coefficient (R^2^) = 0.96. For TP, the assay was linear in the dilution range of 2^0^ to 2^3^ (R^2^ = 0.96), and for anti-HCV was linear in a dilution range of 2^5^ to 2^9^ (R^2^ = 0.98). These determined linear ranges were then used for further experiments.

#### Sensitivity and specificity

Test results of the DPS-based ELISA method using the laboratory basic panel is shown in Table 1. Using the plasma test results as the standard, the sensitivity and specificity of the DPS-based ELISA method for anti-HIV, anti-TP, and anti-HCV were all 100%.

**Table 1:** The analysis of basic plasma panel in laboratory evaluation

(n = 60)			Diagnosis results		Total
			Positive	Negative	
DPS results	HIV	Positive	15	0	60
		Negative	0	45	
	TP	Positive	15	0	
		Negative	0	45	
	HCV	Positive	15	0	
		Negative	0	45	

Sensitivity of DBS against plasma 95.5% (95% CI: 91.3–98,0%). Specificity of DBS against plasma 99.0% (95% CI: 98.1–99.5%)

#### Analytical specificity

Results of testing the DPS-based ELISA method for the laboratory analytical specificity panel is shown in Table 2. The results showed that the method was able to identify coinfections in all of the samples tested, and that coinfections did not interfere with the ability of the method to detect other infections.

**Table 2:** The analysis of plasma panel used to evaluate the capability of analytical specificity

Infection of Sample	DPS results (a/b)			Total
	HIV	TP	HCV	
HIV/TP coinfection	3/3	3/3	0/3	24
HIV/HCV coinfection	3/3	0/3	3/3	
TP/HCV coinfection	0/3	3/3	3/3	
HIV/TP/HCV coinfection	3/3	3/3	3/3	
HBV infection	0/3	0/3	0/3	
HBV/HIV coinfection	3/3	0/3	0/3	
HBV/HCV coinfection	0/3	0/3	3/3	
HBV/TP coinfection	0/3	3/3	0/3	

a means positive number; b means sample number

#### Precision evaluation

Results of the evaluation of the precision of the DPS-based ELISA assay are shown in Table 3. The inter-day, intra-assay, and inter-spot CVs for HIV were 2.10–1.53%, 0.00–3.17% and, 2.84–8.03%, respectively; for TP they were 0.20–12.31%, 0.06–7.25%, and 0.37–11.47%, respectively; for HCV they were 3.01–3.86%, 1.30–5.28%, and 4.18–12.92%, respectively. These results indicate the DPS-based ELISA method provides acceptable precision to screen for HIV, TP, and HCV.

**Table 3:** Evaluation of precision for the DPS-based ELISA method to screen for HIV, TP and HCV(%)

	HIV			TP				HCV		
	15	13	11	3	2	1	0	9	7	5
CV of inter-days	2.10	5.89	1.53	11.36	12.31	6.37	0.20	3.86	3.34	3.01
CV of intra-assay	2.89	3.17	0.00	7.25	4.90	2.76	0.06	5.28	3.51	1.30
CV of inter-spot	8.03	5.41	2.84	11.47	10.23	8.12	0.37	12.92	9.05	4.18

#### Comparison between DPS and plasma specimens

The test result of the 619 matched plasma/DPS samples are shown in Table 4. Of the samples, 25.36% were all 3 agent negative, 17.29% were HIV mono-infected, 16.16% were TP mono-infected, 27.63% were HCV mono-infected, 4.52% were HIV/TP co-infected, 6.79% were HIV/HCV co-infected, 1.13% were HCV/TP co-infected samples, and 1.13%, HIV/HCV/TP co-infected. In the comparison of test results between plasma sample and DPS samples, only 2 pairs of DPS/plasma samples exhibited different results. The DPS-based ELISA method failed to detect 1 HCV mono-infected sample and 1 HIV/HCV/TP co-infected sample. For the HIV/HCV/TP co-infected sample, the discrepant result was for the detection of TP, and the S/CO in the plasma sample was 2.143 and in the DPS sample was 0.5. For HCV, the S/CO was 3.049 in the plasma sample and 0.878 in the DPS sample.

**Table 4:** Comparison analysis of the composition ratio of different infection types based on the results of palsma and DPS samples

Infection type	Plasma	DPS	No. of discrepant results	Discrepancy in
	No. of samples(%)	No. of samples(%)		
Negative samples	157 (25.36)	157 (25.36)	0	
HIV mono-infected	107 (17.29)	107 (17.29)	0	
TP mono-infected	100 (16.16)	100 (16.16)	0	
HCV mono-infected	171 (27.63)	170 (27.46)	1	Plasma + /DBS-
HIV + TP	28 (4.52)	28 (4.52)	0	
HIV + HCV	42 (6.79)	42 (6.79)	0	
HCV + TP	7 (1.13)	7 (1.13)	0	
HIV + HCV + TP	7 (1.13)	6 (1.00)	1	Plasma + /DBS- for TP
Total	619 (100)	617 (99.68)	2 (0.32)	

As shown in Table 5, the proportion of HIV-positive samples, TP-positive samples, and HCV-positive samples were 29.7% (95% confidence interval [CI]: 0.26–0.34), 22.9% (95% CI: 0.20–0.26), and 36.7% (95% CI: 0.33–0.41), respectively. For HIV, the DPS-based method exhibited 100% sensitivity (95% CI: 0.97–1.00) and 100.0% specificity (95% CI: 0.99–1.00). For TP, the DPS-based method had a sensitivity of 98.6% (95% CI: 0.94–1.00) and a specificity of 100% (95% CI: 0.99–1.00), and for HCV the sensitivity was 99.6% (95% CI: 0.97–1.00) and specificity was 100% (95% CI: 0.99–1.00).

**Table 5:** Performance of DPS-based ELISA method to screen for HIV, TP and HCV detection compared with diagnosis results

Screening test	Constituent ratio*	Sensitivity		Specificity		PPV		NPV		Kappa		
	%	95% CI	%	95% CI	%	95% CI	%	95% CI	%	95% CI	coeff	95% CI
Anti-HIV	29.7 (184/619)	0.26–0.34	100.0 (184/184)	0.97–1	100 (435/435)	0.99–1	100.0 (184/184)	0.97–1	100.0 (435/435)	0.99–1	1.00	1.00–1.00
Anti-TP	22.9 (142/619)	0.20–0.26	98.6 (140/142)	0.94–1	100 (477/477)	0.99–1	100.0 (140/140)	0.97–1	99.6 (477/479)	0.98–1	0.99	(0.98–1.00)
Anti-HCV	36.7 (227/619)	0.33–0.41	99.6 (226/227)	0.97–1	100 (392/392)	0.99–1	100.0 (226/226)	0.98–1	99.7 (392/393)	0.98–1	1.00	(0.99–1.00)

* means that the constituent ratio was calculated using diagnosis results. PPV, positive predictive value; NPV, negative predictive value

Take into account the ratios of HIV-, TP-, and HCV-positive samples, the positive predictive value (PPV) of the DPS-based method was 100% for HIV/TP/HCV, and the negative predictive value (NPV) was 100% for HIV, 99.6% for TP, and 99.7% for HCV. The Kappa value between the results of plasma samples and DPS samples was 100% for HIV (*p* < 0.001, 95% CI: 1.00–1.00), 100% for HCV (*p* < 0.001, 95% CI: 0.99–1.00), and 99% for TP (*p* < 0.001, 95% CI: 0.98–1.00).

## Discussion

There has been little research regarding the use of DPS samples to simultaneously screen for anti-HIV, anti-TP, and anti-HCV antibodies [17, 18]. The logistics and storage of DPS cards provides important advantages over the use of liquid forms of blood, such as ease of storage and transportation, and stability of the specimen. The use of DPS sample testing can enable wider screening for HIV, TP, and HCV and help combat the problem of under diagnosis in resource-limited settings.

DPS samples, similar to DBS samples, are drops of plasma collected on filter paper [7]. Because the plasma has been dried on the filter paper, the analyte must first be brought into solution prior to testing. Studies have indicated that the sample volume on the filter paper and how the analyte is eluted from the filter paper and enters solution are important variables for subsequent testing [19–22]. In the present study, we developed a DPS-based ELISA method by determining the optimal conditions for sample elution, and investigated the performance of the assay for simultaneously testing for anti-HIV, -TP, and -HCV antibodies [23].

DPS samples were prepared at 2 plasma volume levels on the filter paper (100 µL and 65 µL) to evaluate the effect of sample volume on the detection of anti-HIV, anti-TP, and anti-HCV antibodies. A plasma volume of 100 µL on the filter paper provided the best results, and this finding is consistent with the results of a prior study that indicated increasing sample quantity was beneficial to the test [24].

To maximizes the efficiency of elution and establish the optimal experiment conditions, we studied the effects of DPS sample size, volume of eluent, elution buffer, duration of elution, and temperature of elution, and determined the optimal values of each [19, 25]. First, we studied the linear range of the DPS-based ELISA method in terms of the detection of HIV, TP, and HCV (Fig. 5). The results indicated that the DPS-based ELISA method exhibited good linearity for the detection of anti-HIV, anti-TP, and anti-HCV antibodies. Optimized intervals were then determined, and found to be 2^14^ to 2^19^, 2^0^ to 2^3^, and 2^5^ to 2^9^ for anti-HIV, anti-TP, anti-HCV antibodies, respectively. Ultimately, we identified the overall optimal parameters for the DPS-based ELISA method for the identification of all 3 agents to be a sample plasma volume of 100 µL, a whole spot size, and eluent volume of 500 µL, an eluent of PBS with 1‰ Tween20 buffer, a duration of elution of 2 h, and an elution temperature of RT.

Unlike traditional samples such as plasma or serum, a DPS sample is a nonstandard diagnostic substance [22]. Therefore, it is necessary to validate the analytical performance of the DPS-based ELISA method, including the sensitivity, specificity, precision, and the anti-jamming compared to that obtained using a plasma sample [26]. Laboratory basic, analytical specificity, and precision panels were constructed to validate the performance of the assay. Compared to the results of plasma testing, the sensitivity and specificity of the DPS-based ELISA method for detection of anti-HIV, anti-TP, and anti-HCV antibodies were all were 100% and 100% (Table 1). As shown in Table 3, the inter-day, intra-assay, and inter-spot CVs for all 3 agents were within the acceptable range (< 15%). These results indicate the performance of the DPS-based ELISA method to screen simultaneously for anti-HIV, anti-TP, and anti-HCV antibodies is comparable to the performance of plasma testing.

Comparison of the results of the testing of 619 matched plasma/DPS samples illustrated that the feasibility of the DPS-based ELISA method to screen simultaneously for anti-HIV, anti-TP, and anti-HCV antibodies [27]. The sensitivity and specificity of the method for detecting HIV were both were 100%, for the detection of TP they were 98.6% and 100%, respectively, and for HCV they were 99.6% and 100%, respectively (Table 5). The DPS-based method failed to detect TP in one sample, and HCV in one sample. This was due to low level of anti-TP and anti-HCV antibodies in the samples (S/CO = 2.143 and 3.049, respectively), but this is relatively uncommon in a clinical situation. The DPS-based ELISA method detected TP and HCV in all other samples.

The Kappa index indicated almost perfect agreement between the DPS-based method results and the plasma testing results for HIV, TP, and HCV. Studies have reported the reliability of ELISA assays using DPS samples for the detection of HAV antibody, HBV antibody/antigen, IP-10 in the diagnosis of tuberculosis infection, and recent HIV-1 infection [12, 28]. Our study showed that a DPS-based ELISA method can reliably simultaneously screen for HIV, TP, and HCV. Consequently, it may facilitate detection of HIV, TP, and HCV infection in settings where access to screening is difficult due to underdeveloped laboratory facilities and geographic inaccessibility, and potentially it can also be available for a referral system in hospital [24].There are some limitations to this study that should be acknowledged. The number of samples used was relatively small, and the study used stored samples rather than those that were newly collected. In addition, the study was limited to samples collected in a specific geographic region and from a specific population. The use of different testing kits may provide different results, and we did not examine the effect of storage time or temperature of the DPS samples on the results.

DPS and DBS are both techniques used for collecting and preserving biological fluid samples, employed for diagnosing and monitoring various diseases [3, 7, 10, 12]. While they share some similarities, there are also key differences. Although DPS samples may be less cumbersome to handle compared to DBS, as they don't require red blood cell separation as DBS samples do, DPS has its unique advantages. Firstly, DPS is prepared from plasma, making the samples purer, containing molecules and biomarkers from the plasma. This makes DPS samples more suitable for certain molecular or serum testing since they lack red blood cells and other components that could interfere with or mask the detection of specific biomarkers. Secondly, plasma in DPS samples is typically more stable, while the components in DBS samples may not be as stable as DPS and require stricter temperature control to maintain their integrity over time, or they may degrade. Furthermore, plasma in DPS samples is already inactivated, resulting in lower biological risk during handling and transportation, while DBS samples might contain active pathogens, necessitating more stringent biosafety measures. In certain situations, DBS samples may be more appropriate, while in others, DPS samples may offer advantages. Both have their strengths and weaknesses, especially in resource-limited or remote areas. This study is focused on the feasibility of using DPS as an alternative sample within the context of the HIV sentinel testing network in China.

## Conclusion

In conclusion, we developed a DPS-based ELISA method to simultaneously screen for HIV, TP, and HCV, and showed the performance of the method was similar to that of plasma testing for all 3 agents. This method can allow testing in difficult to access regions and in LMICs or resource-limited settings.

## Data Availability

Not applicable. All relevant data are within the paper and its Supporting Information fles.
